# Future Challenges and Critical Approach to Metrology in Patients with Axial Spondyloarthritis

**DOI:** 10.3390/diagnostics11091533

**Published:** 2021-08-25

**Authors:** Juan L. Garrido-Castro, Eduardo Collantes-Estévez, Francisco Alburquerque-Sendín, Clementina López-Medina

**Affiliations:** 1 Maimonides Biomedical Research Institute of Cordoba, 14004 Córdoba, Spain; cc0juanl@uco.es (J.L.G.-C.); eduardo.collantes.sspa@juntadeandalucia.es (E.C.-E.); clementinalopezmedina@gmail.com (C.L.-M.); 2 Department of Rheumatology, University Hospital Reina Sofía, 14004 Córdoba, Spain; 3 Department of Medical and Surgical Sciences, University of Córdoba, 14004 Córdoba, Spain; 4 Department of Nursing, Pharmacology and Physical Therapy, University of Córdoba, 14004 Córdoba, Spain

Axial spondyloarthritis (axSpA) is a rheumatic inflammatory chronic disease that mainly affects the spine, producing inflammation and structural damage at the vertebral level (erosions, syndesmophytes, and bony bridges). This leads to a reduction of mobility in axSpA patients [1] that requires assessment by rheumatologists to closely monitor patients and analyse the efficacy of prescribed treatments.

Several tools are used for the evaluation of these patients [2]; thus, self-assessed patient report outcomes (PRO) questionnaires may be completed by the patient to evaluate function (Bath Ankylosing Spondylitis Functional Index—BASFI), whereas other tools monitor disease activity (Bath Ankylosing Spondylitis Disease Activity Index—BASDAI) and the patient’s quality of life (Assessment of SpondyloArthritis International Society Health Index—ASAS-HI), among others. In addition, other indexes are used to analyse radiographic structural damage (modified Stoke Ankylosing Spondylitis Spinal Score—mSASSS) and activity indexes based on blood tests (Ankylosing Spondylitis Disease Activity Score—ASDAS).

After pain, the most common concern of patients is loss of spinal mobility, due to its impact on function. This is commonly evaluated using mobility tests based on measuring tapes and goniometers, which were defined over three decades ago: the Schöber test, cervical rotation, tragus to wall distance, lateral flexion, intermalleolar distance, etc. Metrological indexes have also been defined based on some of these measurements, such as the BASMI index (Bath Ankylosing Spondylitis Metrology Index) [2]. Furthermore, several of these are indirect measures, such as the fingertips to floor distance, which reflects the mobility of the spinal joints at the vertebral level, but also includes hip and shoulder motion, whereas others, such as trunk lumbar rotation, are impossible to obtain using these tools. The assessment of mobility in axSpA patients represents one of the most important measurements that a rheumatologist should conduct in routine practice consultations. Indeed "limited range of motion of the lumbar spine in both forward and lateral bending" was used in the original diagnostic criteria (New York criteria) of axSpA, although this concept is not frequently used. Furthermore, the benefit of mobility measurements is that they provide an objective value, unlike PROs, moreover they are a faithful reflection of inflammation and radiographic structural damage (without radiating the patient).

Despite its importance, due to the significant variability and observer dependence of these classical mobility measures [3,4], mobility has not been used as a biomarker in recent treatment efficacy studies or in clinical trials. In the short term, improvements in mobility are small and conventional metrology is unable to detect these changes. This context highlights the need to use advanced technological tools that are able to detect these small changes using more accurate systems, that are not observer-dependent, and that provide more detailed information on patient spinal mobility.

## 1. Advanced Metrology Tools for Measuring Spinal Mobility in axSpA

With the aim of introducing novel approaches for the assessment of mobility in axSpA, various initiatives have been carried out in recent times, such as the use of technology. Physiotherapists now use many simple tests, such as functional tests, including the 30 s sit to stand, timed up and go test, 6/10 m walk test, etc. However, these tests are not commonly used to evaluate mobility in axSpA [5], perhaps because the main interest is to assess spinal mobility, however this indirectly affects other functions such as gait ability. Few studies have analysed gait in axSpA [6,7], although such assessments are commonly used in other areas of physiotherapy. Some authors have designed their own functional tests, involving putting on socks or getting up from the floor, with good results in the evaluation of therapies [8]. Swinnen et al. proposed to automate and objectify the functional BASFI test, introducing the use of an accelerometer (a small electronic sensor that measures the acceleration forces acting on an object, in order to determine object movement, speed, and orientation) and specific algorithms [9]. Snow et al. [10], compared concurrent criteria validity of a tri-axial accelerometer compared with a conventional lumbar mobility test: original and modified Schöber and lateral flexion and radiographic assessments, with the accelerometer providing better results in forward bending. Kiefer et al. [11] tested a specific electromagnetic device, the Epionics SPINE (Epionics Medical GmbH, Potsdam, Germany) for measuring lumbar and thoracic mobility in axSpA, with good validity results, although this device is only designed for the back and not for neck mobility, which is also affected in axSpA.

Several studies have used motion capture applied to spinal mobility in these patients [12]. This technique is widely used in other fields such as sports performance and the animation industry. It has a high level of accuracy and reliability, although it requires a dedicated motion analysis laboratory and this is not always easily available at a hospital. To try to design a system that is more feasible and portable, some studies have used portable inertial sensors (Inertial Measurement Unit—IMU) [13] which are beginning to be used in physiotherapy clinics. These portable sensors are based on accelerometers, gyroscopes, and magnetometers, which combine their data using a fusion algorithm producing the orientation of the sensors in terms of quaternions or Euler angles (pitch, yaw, roll). These sensors have also been used to measure the patient’s physical activity during controlled tests and over 24 h to analyse the patient’s activities of daily living as a biomarker [14,15,16,17].

Some metrology indexes have been introduced, such as the BASMI, using advanced technological tools. The UCOASMI [18] is a metrology index based on three plane ranges of spinal movement obtained by motion capture. A similar index was defined by using measures obtained by an IMU sensor-based system, the IMUASMI [19]. These indexes have a good correlation with BASMI and with other axSpA outcomes although with better reliability, accuracy, and responsiveness features compared to BASMI (Table 1).

## 2. Physical Therapy, Rehabilitation, and New Tools

As previously reported, there are still few evidence-based tools for assessment and diagnostic purposes in the clinical setting of physical therapy and rehabilitation. Furthermore, most tools have been developed for other clinical areas, such as rheumatology or nursing, with similar, although slightly different aims to those of physiotherapy [20]. Moreover, the main barriers encountered by physiotherapists for the application of standardized measures as recommended in clinical practice guidelines include lack of knowledge, lack of time, their availability and the lack of management support, among others [21]. Thus, more accurate tools could be applied if they were fast and easy to use, correctly described and if the quality and applicability of their results were improved.

Some of the current tools used in the assessment of patients with axial spondyloarthritis, despite being developed in other disciplines, may be applicable for physical therapy and rehabilitation settings. Thus, BASMI, BASFI and BASDAI have been successfully applied as outcomes in the rehabilitation of axial spondyloarthritis [22]. It is expected that new technological tools can improve the evaluation and diagnosis in physiotherapy and rehabilitation, although an in-depth determination of the metric features is required before these may be applied. Some of these new tools are related to spinal and hip movement (motion analysis optical systems, IMUs), instrumented functional tests (EMG, function, and quality of movement), specific properties of soft tissues (myotonometry, elastography, sonography), daily physical therapy (wearables, mobile apps), which are commonly assessed in the clinical setting of physical therapy.

## 3. Future Perspectives of Metrology in axSpA

Conventional metrology instruments must be improved with the inclusion of new technologies; however, these must be simple, reproducible, and quick to perform, in order to be applicable during daily clinical practice. Nevertheless, when the complexity of the tools hinders their inclusion in daily practice, their applicability in research and innovation fields should be considered. There is a need for the standardization of indices based on measurements obtained by these systems, so that various manufacturers can design sensors and equipment that provide the same measurement, independent of the system used. Some steps have been taken in this direction; however, they are insufficient.

In summary, the implementation these new technological tools in the clinical daily practice of physiotherapy continues to pose a challenge for clinicians.

## Figures and Tables

**Table 1:** Technical alternatives to assess patient mobility in axSpA.

	Activity Monitoring	Spinal Mobility	Functional Tests	Gait Analysis	
Motion Capture		X		X	Reflective markers placed on the patient’s back are detected and located (X,Y,Z) by infrared cameras. Based on these positions, the inclination of segments and planes is obtained.
Inertial sensors	X	X	X	X	Independent wearable sensors are located in the spine. These devices provide the real time orientation of each sensor. Relative angles between sensors are also calculated.
Accelerometer	X		X		The sensor obtains acceleration in the three axes (X,Y,Z) that can detect strong or weak physical activity. They can also provide orientation for identifying several tasks.
